# Development and Validation of a Novel Lymphedema Monitoring System Using a Bodysuit and a Smartphone: Prospective Comparative Study

**DOI:** 10.2196/77935

**Published:** 2026-03-06

**Authors:** Ryo Karakawa, Hidehiko Yoshimatsu, Katsu Karakawa, Kuniko Utsugi, Kengo Ono, Eri Kojima, Takayasu Yamada, Duncan Thomas Eason, Francois Jean Leon Goffinet, Tomoyuki Yano

**Affiliations:** 1 Cancer Institute Hospital of the Japanese Foundation for Cancer Research Tokyo Japan; 2 Department of Plastic and Reconstructive Surgery Memorial Sloan Kettering Cancer Center New York, NY United States; 3 Kawabata Lymphedema Clinic Kobe Japan; 4 ZOZO, Inc Chiba Japan; 5 ZOZO New Zealand Limited Auckland New Zealand

**Keywords:** measurements, limbs, smartphone, bodysuit, lymphedema, patients

## Abstract

**Background:**

Accurate limb circumference measurement is essential for the diagnosis and management of lymphedema, facilitating the objective evaluation of treatment outcomes and early detection of disease progression. Although manual tape measurements are inexpensive and widely used, they are limited by interobserver variability and low reproducibility. Advanced modalities such as computed tomography and magnetic resonance imaging provide high precision but are costly and impractical for routine or home-based use. To overcome these limitations, we developed and validated a novel 3D measurement system that integrates a marker-based bodysuit (ZOZOSUIT2) with a smartphone app, enabling fast, accurate, and reproducible limb circumference assessment.

**Objective:**

This study aimed to evaluate the accuracy, repeatability, and time efficiency of the 3D measurement bodysuit and smartphone system compared with those of manual tape measurements in patients with upper and lower limb lymphedema.

**Methods:**

This prospective study included 26 patients (n=10 with upper limb lymphedema and n=16 with lower limb lymphedema). Using the ZOZOSUIT2, which contains approximately 20,000 dot markers, 12 full-body photographs were captured with a smartphone, and a 3D model was automatically generated. Circumference values were extracted at 10 standardized points on the upper limb (from the wrist to the top of the upper arm) and 9 on the lower limb (from the ankle to the base of the thigh). Each measurement was repeated 3 times to assess repeatability. Manual tape measurements were used as the reference standard. For each measurement point, the repeatability (intrameasurement variation) and the mean absolute error between the 2 methods were calculated. Measurement time was also recorded for both methods and compared statistically using the Wilcoxon signed-rank test.

**Results:**

Across all measurement points, the median repeatability of the bodysuit smartphone system ranged from 2.2 to 6.4 mm, indicating high reproducibility. When compared with manual measurements, the median of the mean absolute error for upper limb points ranged from 6.7 to 20.5 mm, and for lower limb points ranged from 5.5 to 15.9 mm. Relatively larger discrepancies were observed at the wrist (median 19.8, IQR 15.5-24.1 mm), 5 cm proximal to the wrist (median 20.5, IQR 8.5-28.1 mm), and the base of the thigh (median 15.9, IQR 10.2-25.9 mm). Mean measurement time was significantly shorter with the bodysuit smartphone system (88.2, SD 16.0 seconds) than with manual tape measurement (293.8, SD 58.5 seconds; *P*<.001).

**Conclusions:**

The bodysuit smartphone system enables rapid, precise, and highly reproducible limb circumference assessment in patients with lymphedema. Despite minor differences at anatomical sites with complex contours, such as the wrist and the thigh base, the overall accuracy and time efficiency were clinically acceptable. This system may serve as a practical and scalable solution for both clinical and home-based lymphedema monitoring, contributing to the objective and standardized assessment of limb volume.

## Introduction

Lymphedema is a chronic condition characterized by the abnormal accumulation of lymphatic fluid in the interstitial tissues, often resulting in swelling, discomfort, and functional impairments [1]. Accurate measurement of limb circumference is critical for both the diagnosis and monitoring of lymphedema, as well as for evaluating the effectiveness of conservative therapies or surgical interventions [2]. Early detection through precise measurement is particularly important to mitigate disease progression and optimize therapeutic outcomes [3].

Various methods for limb circumference measurement have been developed, each with distinct advantages and limitations. Manual circumference measurement using a tape measure is widely used because of its simplicity and low cost. However, it is inherently prone to interobserver and intraobserver variability and provides limited data points, restricting its utility in comprehensive assessment [4]. Advanced imaging techniques such as computed tomography and magnetic resonance imaging offer high accuracy and detailed data; however, their high cost, limited accessibility, and invasive nature make them unsuitable for routine or home-based monitoring [5,6]. These limitations underscore the need for a measurement method that is accurate, noninvasive, cost-effective, and user-friendly.

In recent years, technological advancements have facilitated the use of 3D scanning and smartphone-based tools for health monitoring. These innovations have made it possible to obtain detailed and accurate measurements with minimal invasiveness, providing new opportunities for disease management in both clinical and home settings [7,8]. However, there remains a lack of practical solutions that combine accuracy, user-friendliness, and cost-effectiveness, particularly for conditions such as lymphedema that require frequent and precise monitoring. To address this challenge, we have developed a novel approach using a 3D measurement bodysuit (ZOZOSUIT2; ZOZO Inc) and a smartphone [9]. With a standard smartphone camera, participants could generate a 3D virtual representation of their bodies in a few minutes. The primary objective of this study was to validate the accuracy and reliability of this newly developed method for measuring limb circumference in patients with lymphedema.

## Methods

### Study Design and Participants

This was a prospective, multicenter observational study conducted to validate the accuracy and reliability of limb circumference measurements using a 3D measurement bodysuit (ZOZOSUIT2) and a smartphone. Consecutive patients diagnosed with upper or lower limb lymphedema who visited the Cancer Institute Hospital between November 2021 and February 2023 were screened for eligibility. Patients were identified from the outpatient clinic records and were approached in person during their regular visits. Participants who met the inclusion criteria and provided written informed consent were enrolled. Those who declined participation or were unable to wear the designated bodysuit due to size incompatibility were excluded.

### Ethical Considerations

This study was reviewed and approved by the institutional review board of the Japanese Foundation for Cancer Research (2021-GA-1050) on April 5, 2022. All participants provided written informed consent before enrollment after receiving a full explanation of the study objectives and procedures. All collected data were anonymized before analysis to protect participant privacy and confidentiality. No financial or other compensation was provided to participants for their involvement in the study. This study was conducted and reported in accordance with the STROBE (Strengthening the Reporting of Observational Studies in Epidemiology) guidelines [10].

### Data Collection and Manual Measurement

Patient characteristics and clinical data were obtained, including age, sex, BMI, and clinical stages based on the International Society of Lymphology (ISL) classification. Manual circumference measurements were performed using a standard measuring tape for comparison. For the upper limbs, circumferences were measured at the following 10 predefined points: top of the upper arm, 10 cm proximal to the elbow, midpoint of the upper arm, 5 cm proximal to the elbow, the elbow, 5 cm distal to the elbow, maximum point of the forearm, 10 cm proximal to the wrist, 5 cm proximal to the wrist, and the wrist. For the lower limbs, circumferences were measured at the following 9 predefined points: base of the thigh, 20 cm proximal to the knee, 10 cm proximal to the knee, the knee, just below the knee, 5 cm distal to the knee, maximum point of the lower leg, 10 cm distal to the knee, and the ankle.

To ensure consistency between methods, manual tape measurements were performed in a standing position, matching the posture used for the bodysuit smartphone measurements. In addition to circumference measurements, the time required to complete manual measurements for both upper and lower limbs was recorded. Measurement time was defined as the total duration from placement of the measuring tape at the first predefined point to completion of the final measurement.

### Measurement System and Procedure

**Figure 1:**
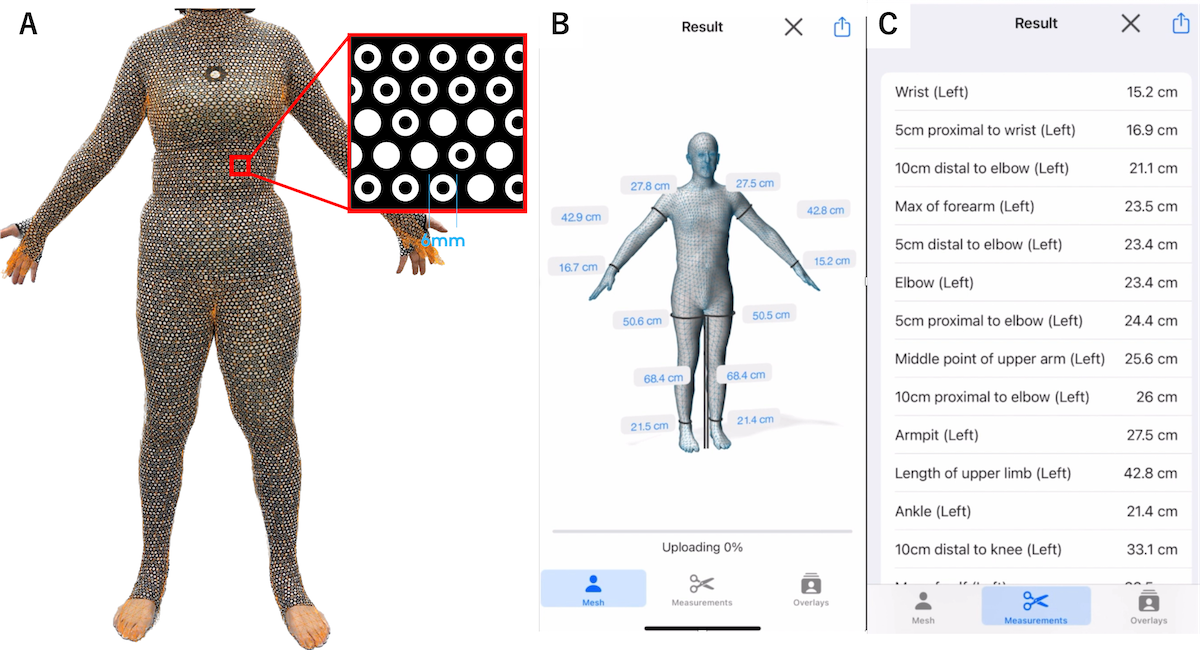
Workflow of the 3D measurement system using the ZOZOSUIT2 and smartphone app. (A) The ZOZOSUIT2 is a bodysuit embedded with approximately 20,000 dot markers, enabling high-accuracy 3D measurement through a smartphone camera. (B) The application analyzes the 12 images to extract the 2D position of each visible marker and the participant’s silhouette, and the 3D virtual human body model is displayed in the app. (C) The images were then processed by proprietary software to generate a 3D model of the participant’s body, from which limb circumferences were automatically extracted at multiple points.

The ZOZOSUIT2 is a bodysuit embedded with approximately 20,000 dot markers, enabling high-accuracy 3D measurement through a smartphone camera (Figure 1). Participants wore the ZOZOSUIT2, and 12 photographs were taken from different angles using a smartphone (iPhone 14; Apple Inc) according to the manufacturer’s protocol.

The participants were instructed to adopt a posture with their arms and lower limbs slightly spread out. The smartphone running the fitting app was placed on a phone stand at a height of 70 cm and positioned 1.5 m away from the participant. The app used audio prompts to guide the participant. Participants were instructed to turn clockwise by 30° every 6 seconds. Participants actively rotated their own body position without the use of a revolving platform. The app automatically captured a new image at the end of each interval. At the end of the whole process, the app had acquired 12 images of the participant. The app analyzed the 12 images to extract the 2D position of each visible marker and the participant’s silhouette. Finally, a 3D virtual human body model was displayed in the app. These images were processed by proprietary software to generate a 3D model of the participant’s body, from which limb circumferences were automatically extracted at multiple points (Figure 1). The measurement time was recorded.

### Statistical Analyses

To assess the reproducibility of the bodysuit smartphone system, 3 consecutive measurements were performed for each participant under identical conditions. Repeatability for each measurement point was defined as the mean of the absolute differences between paired measurements (scan 1 vs scan 2, scan 2 vs scan 3, and scan 1 vs scan 3) across all participants. The limb circumference measurements obtained with the ZOZOSUIT2 and smartphone were compared to those obtained through manual measurements using a tape measure. The mean absolute error for each measurement point was calculated to evaluate accuracy.

Measurement times were recorded for both the bodysuit smartphone method and manual measurements. The start time was defined as the point at which the measurement process began (putting on the bodysuit or preparing the tape measure), and the end time was when all measurements were completed. Statistical comparisons of measurement times were performed between the 2 methods.

All statistical analyses were conducted using Google Sheets (Google LLC). Built-in spreadsheet functions were used to calculate descriptive statistics (mean, SD), and paired *t* tests (2-tailed) were performed to compare measurement times between the bodysuit smartphone and manual methods. Statistical significance was set at *P*<.05. The data were cross-checked using Microsoft Excel (version 16.106.2; Microsoft Corp) to verify calculation consistency.

## Results

### Patient Characteristics

Our study included 26 patients, consisting of 10 with upper limb lymphedema and 16 with lower limb lymphedema. We performed a total of 78 measurements using the bodysuit smartphone method. Table 1 shows the demographics of patients with upper limb lymphedema. The mean age was 56.1 (SD 15.2; range 30-79) years. All patients were female. The mean BMI was 22.6 (SD 2.3; range 17.9-25.9) kg/m^2^. The causative disease of lymphedema was breast cancer in all patients. Of 10 patients, lymphedema severity was stage I in 3 (30%), stage IIa in 2 (20%), and stage IIb in 5 (50%), based on the ISL classification.

**Table 1:** Demographics of patients with upper limb lymphedema (N=10).

Parameter			Value
Age (years), average (SD; range)			56.1 (15.2; 30-79)
**Sex, n (%)**			
	Female	10 (100)	
**Physical parameters, average (SD; range)**			
	Height (cm)	155.2 (5.8; 146-163)	
	Weight (kg)	54.8 (8.4; 43.0-68.8)	
	BMI (kg/m^2^)	22.6 (2.3; 17.9-25.9)	
**Cause of disease, n (%)**			
	Breast cancer	10 (100)	
**International Society of Lymphology stage, n (%)**			
	I	3 (30)	
	IIa	2 (20)	
	IIb	5 (50)	

Table 2 shows the demographics of patients with lower limb lymphedema. The mean age was 60.9 (SD 11.2; range 43-76) years. All patients were female. The mean BMI was 21.8 (SD 2.8; range 16.5-28.1) kg/m^2^. Of 16 patients, the causative disease of lymphedema was cervical cancer in 9 (56%), uterine cancer in 5 (31%), and ovarian cancer in 2 (13%). Lymphedema severity was stage I in 2 (13%), stage IIa in 5 (31%), and stage IIb in 9 (56%), based on the ISL classification.

**Table 2:** Demographics of patients with lower limb lymphedema (N=16).

Parameter			Value
Age (years), average (SD: range)			60.9 (11.2; 43-76)
**Sex, n (%)**			
	Female	10 (100)	
**Physical parameters, average (SD: range)**			
	Height (cm)	157.8 (5.4; 148-167)	
	Weight (kg)	54.2 (6.4; 45.6-65.5)	
	BMI (kg/m^2^)	21.8 (2.8; 16.5-28.1)	
**Cause of disease, n (%)**			
	Cervical cancer	9 (56)	
	Uterine cancer	5 (31)	
	Ovarian cancer	2 (13)	
**International Society of Lymphology stage, n (%)**			
	I	2 (13)	
	IIa	5 (31)	
	IIb	9 (56)	

### Repeatability of Limb Circumference Measurements

**Figure 2:**
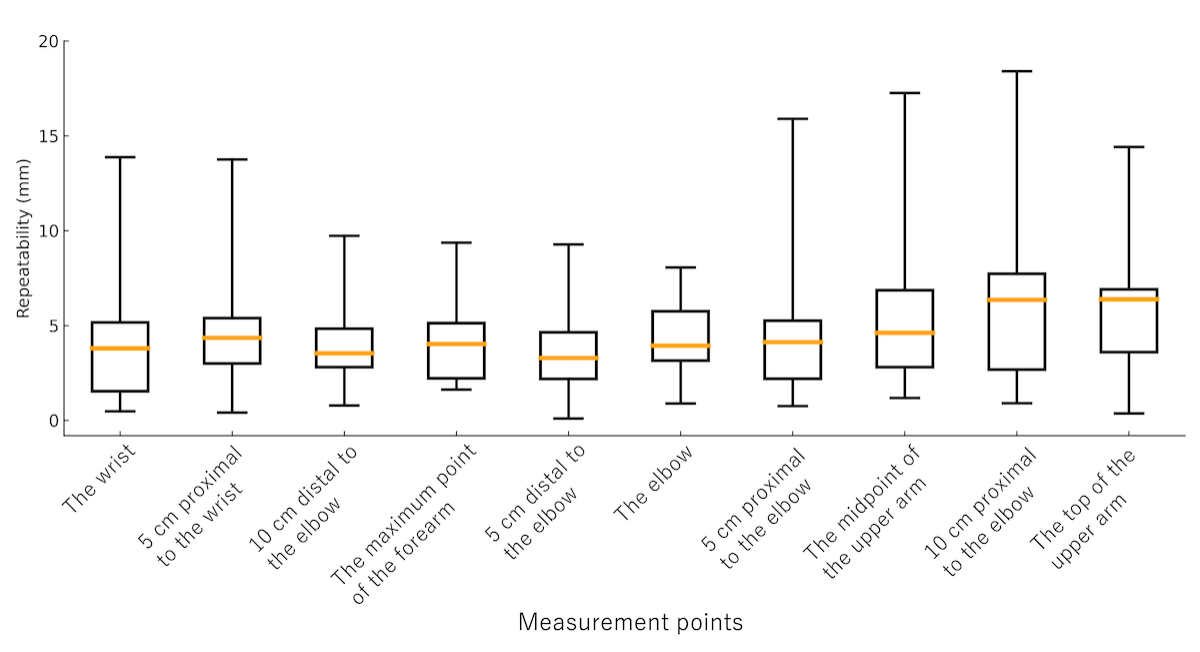
Repeatability of measurements at each upper limb point. Box-and-whisker plots show the repeatability of circumference measurements at 10 predefined upper limb points obtained using the 3D measurement bodysuit smartphone system. Each box represents the IQR, the orange line indicates the median, and the whiskers show the full range. Measurement points include the wrist, 5 cm proximal to the wrist, 10 cm distal to the elbow, the maximum point of the forearm, 5 cm distal to the elbow, the elbow, 5 cm proximal to the elbow, the midpoint of the upper arm, 10 cm proximal to the elbow, and the top of the upper arm.

**Figure 3:**
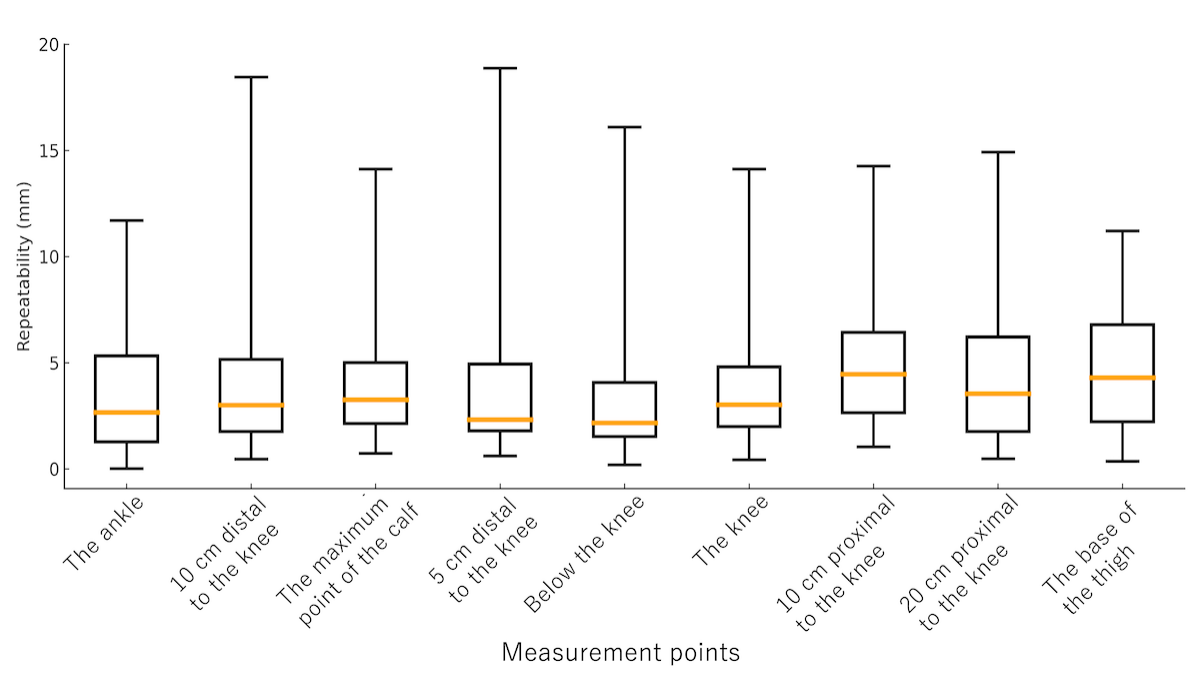
Repeatability of measurements at each lower limb point. Box-and-whisker plots show the repeatability of circumference measurements at 9 predefined lower limb points obtained using the 3D measurement bodysuit smartphone system. Each box represents the IQR, the orange line indicates the median, and the whiskers show the full range. Measurement points include the ankle, 10 cm distal to the knee, the maximum point of the calf, 5 cm distal to the knee, just below the knee, the knee, 10 cm proximal to the knee, 20 cm proximal to the knee, and the base of the thigh.

The median repeatability of limb circumference measurements obtained using the bodysuit smartphone system ranged from 2.2 to 6.4 mm across all measurement points for both the upper and lower limbs. Specifically, the median repeatability values for the upper limbs were as follows: the wrist, 3.8 (IQR 1.5-5.2) mm; 5 cm proximal to the wrist, 4.4 (IQR 3.0-5.4) mm; 10 cm distal to the elbow, 3.5 (IQR 2.8-4.8) mm; the maximum point of the forearm, 4.0 (IQR 2.2-5.1) mm; 5 cm distal to the elbow, 3.3 (IQR 2.2-4.6) mm; the elbow, 3.9 (IQR 3.2-5.8) mm; 5 cm proximal to the elbow, 4.1 (IQR 2.2-5.3) mm; the midpoint of the upper arm, 4.6 (IQR 2.8-6.9) mm; 10 cm proximal to the elbow, 6.4 (IQR 2.7-7.7) mm; and the top of the upper arm, 6.4 (IQR 3.6-6.9) mm (Figure 2). For the lower limbs, the median values were as follows: the ankle, 2.7 (IQR 1.3-5.3) mm; 10 cm distal to the knee, 3.0 (IQR 1.8-5.2) mm; the maximum point of the lower leg, 3.3 (IQR 2.1-5.0) mm; 5 cm distal to the knee, 2.3 (IQR 1.8-4.9) mm; just below the knee, 2.2 (IQR 1.5-4.1) mm; the knee, 3.0 (IQR 2.0-4.8) mm; 10 cm proximal to the knee, 4.5 (IQR 2.6-6.4) mm; 20 cm proximal to the knee, 3.5 (IQR 1.8-6.2) mm; and the base of the thigh, 4.3 (IQR 2.2-6.8) mm (Figure 3). All repeatability values were below 10 mm, indicating a high level of reproducibility and confirming the system’s reliability for both upper and lower limb measurements.

### Comparison Between the Bodysuit Smartphone Method and Manual Measurements

**Figure 4:**
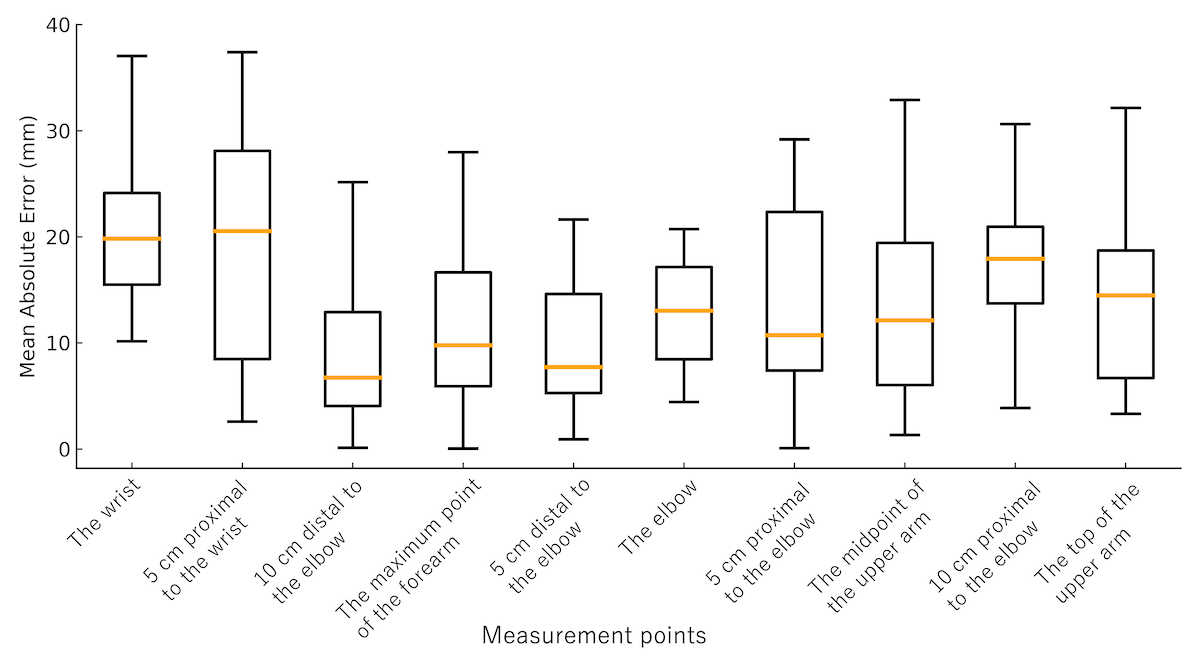
Mean absolute error between manual and bodysuit smartphone measurements at each upper limb point. Box-and-whisker plots illustrate the mean absolute error between manual and digital measurements across 10 measurement points on the upper limb (the wrist, 5 cm proximal to the wrist, 10 cm distal to the elbow, the maximum point of the forearm, 5 cm distal to the elbow, the elbow, 5 cm proximal to the elbow, the midpoint of the upper arm, 10 cm proximal to the elbow, and the top of the upper arm). The boxes represent the IQR, the central line indicates the median, and the whiskers denote the range excluding outliers. The overall median of the mean absolute error ranged from 6.7 to 20.5 mm, with higher variability observed in the proximal regions.

The mean absolute error between manual measurements and those obtained using the bodysuit smartphone system for the upper limb is shown in Figure 4. The median of the mean absolute error values across all upper limb measurement points ranged from 6.7 to 20.5 mm. Specifically, the median of the mean absolute error values were as follows: the wrist, 19.8 (IQR 15.5-24.1) mm; 5 cm proximal to the wrist, 20.5 (IQR 8.5-28.1) mm; 10 cm distal to the elbow, 6.7 (IQR 4.1-12.9) mm; the maximum point of the forearm, 9.8 (IQR 5.9-16.7) mm; 5 cm distal to the elbow, 7.7 (IQR 5.3-14.6) mm; the elbow, 13.0 (IQR 8.5-17.2) mm; 5 cm proximal to the elbow, 10.8 (IQR 7.4-22.4) mm; the midpoint of the upper arm, 12.1 (IQR 6.0-19.4) mm; 10 cm proximal to the elbow, 17.9 (IQR 13.7-21.0) mm; and the top of the upper arm, 14.5 (IQR 6.7-18.7) mm.

**Figure 5:**
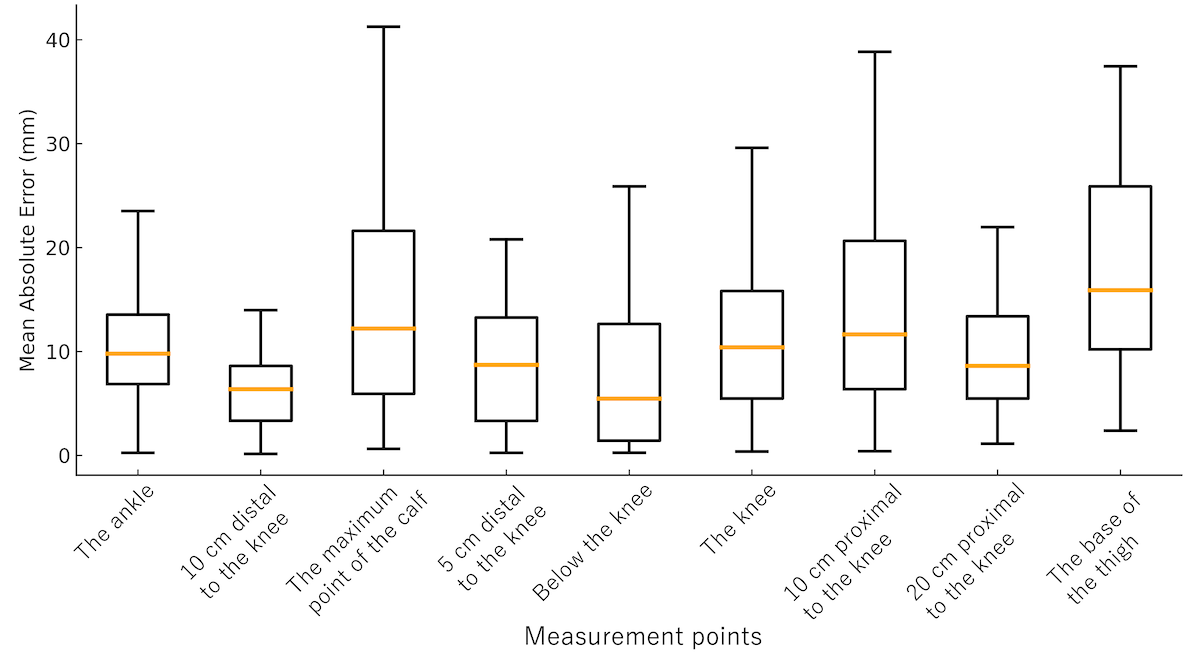
Mean absolute error between manual and bodysuit smartphone measurements at each lower limb point. Box-and-whisker plots illustrate the mean absolute error between manual and digital measurements across 9 measurement points on the lower limb (the ankle, 10 cm distal to the knee, the maximum point of the calf, 5 cm distal to the knee, just below the knee, the knee, 10 cm proximal to the knee, 20 cm proximal to the knee, and the base of the thigh). The boxes indicate the IQR, the central line shows the median, and the whiskers represent the range. The overall median of the mean absolute error ranged from 5.5 to 15.9 mm, with slightly larger deviations observed at the base of the thigh.

The mean absolute error between manual measurements and those obtained using the bodysuit smartphone system for the lower limb is shown in Figure 5. The median of the mean absolute error values ranged from 5.5 to 15.9 mm across all measurement points. Specifically, the median of the mean absolute error values were as follows: the ankle, 9.8 (IQR 6.9-13.6) mm; 10 cm distal to the knee, 6.4 (IQR 3.3-8.6) mm; the maximum point of the calf, 12.2 (IQR 5.9-21.6) mm; 5 cm distal to the knee, 8.7 (IQR 3.3-13.3) mm; just below the knee, 5.5 (IQR 1.4-12.7) mm; the knee, 10.4 (IQR 5.5-15.8) mm; 10 cm proximal to the knee, 11.6 (IQR 6.4-20.7) mm; 20 cm proximal to the knee, 8.6 (IQR 5.5-13.4) mm; and the base of the thigh, 15.9 (IQR 10.2-25.9) mm.

Furthermore, compared to the manual measurement group, the bodysuit smartphone method group demonstrated significantly shorter mean measurement times (88.2, SD 16.0 vs 293.8, SD 58.5 seconds; *P*<.001). The variability in measurement time was also considerably lower in the bodysuit smartphone method group than in the manual measurement group, indicating a more consistent measurement process.

## Discussion

This study aimed to validate the accuracy and reliability of a novel limb circumference measurement system using a 3D measurement bodysuit and a smartphone compared to the conventional manual measuring tape method. The findings demonstrate that the bodysuit smartphone system is not only highly accurate and reproducible but also significantly more time-efficient than manual methods, indicating its potential as a valuable tool for lymphedema management.

The median of the mean absolute error across all measurement points for the 3D measurement bodysuit smartphone system was less than 10 mm. This level of accuracy is within clinically acceptable thresholds for lymphedema monitoring, where errors of less than 10 mm are generally considered sufficient to detect changes in limb circumference and disease progression [11]. Furthermore, the reproducibility of this method suggests transformative potential for the way limb circumference measurements are made. Unlike traditional manual measurements, which require the assistance of health care professionals or trained individuals, this method enables patients to perform measurements independently at home. This autonomy could significantly reduce the burden on health care systems by minimizing the need for clinic visits, thereby conserving medical resources and streamlining patient care [7]. The implications of these findings highlight the potential of the 3D measurement bodysuit smartphone system to revolutionize lymphedema management through improved accessibility and efficiency.

When comparing the mean absolute error between manual measurements and those obtained using the 3D measurement bodysuit smartphone system, the errors at most measurement points were less than 20 mm. This level of accuracy is considered clinically acceptable and comparable to manual measurements. However, in the upper limb, relatively larger errors were observed at the wrist and 5 cm proximal to the wrist, with median of the mean absolute errors of 19.8 (IQR 15.5-24.1) mm and 20.5 (IQR 8.5-28.1) mm, respectively.

In the lower limb, the base of the thigh also showed a relatively larger deviation, with median of the mean absolute errors of 15.9 (IQR 10.2-25.9) mm. These differences are likely attributable to the difficulty of obtaining consistent manual measurements at these anatomical sites rather than limitations of the bodysuit smartphone system itself. The thigh base includes a large and deformable soft tissue volume, which makes it challenging to define and reproduce an identical measurement level with a tape measure, particularly in the standing position. At the wrist, bony prominences such as the radial and ulnar styloid processes can interfere with stable tape placement. Considering the high repeatability of the bodysuit based method, it may offer greater reproducibility and reduce variability caused by individual measurers, thus contributing to the standardization of limb circumference measurement. These features highlight the potential of this method to provide consistent and reliable data for both clinical practice and research settings. In addition, posture may also influence limb circumference measurement because gravity affects lymphatic fluid distribution. In our study, both manual and bodysuit smartphone measurements were performed in a standing position to minimize posture-related discrepancies. This standardized posture helped ensure that any differences between the 2 methods were due to measurement technique rather than variation in limb fluid distribution.

In addition to its accuracy, the 3D measurement bodysuit smartphone system demonstrated significantly shorter measurement times than manual methods. The bodysuit based method required an average of 88.2 (SD 16.0) seconds, whereas manual measurements took 293.8 (SD 58.5) seconds on average. This time efficiency has important implications for clinical workflows, particularly in high-volume lymphedema clinics. By reducing the time needed for circumference measurements, health care providers can focus more on other critical aspects of patient care such as treatment planning and counseling. This efficiency underscores the potential of this system to streamline lymphedema management in both clinical and home settings.

Advanced imaging modalities such as magnetic resonance imaging and computed tomography are capable of providing detailed anatomical and volumetric data, making them highly accurate tools for lymphedema assessment. However, their high cost, limited accessibility, and the need for specialized facilities and personnel render them impractical for routine use or home-based monitoring. The 3D measurement bodysuit smartphone system offers a noninvasive, portable, and cost-effective alternative that bridges the gap between these advanced imaging techniques and traditional manual methods. Additionally, devices such as the Perometer (Pero-System GmbH), which use infrared light to measure limb volume, have been established as reliable tools in clinical settings. While the Perometer is less labor intensive and provides high precision, its reliance on bulky, fixed equipment and higher costs limit its applicability for home use or resource-limited environments [12,13]. In contrast, the bodysuit smartphone system leverages widely available smartphone technology, thereby providing greater accessibility and usability. Its portability and ease of use make it particularly suitable for frequent or remote monitoring, empowering patients and reducing the burden on health care providers. This combination of practicality and accuracy positions it as a promising alternative for routine lymphedema management.

The implications of these findings are significant. Early detection and timely intervention are crucial for preventing lymphedema progression, and the 3D measurement bodysuit smartphone system enables frequent and accurate monitoring, empowering patients to take an active role in their care. By facilitating self-monitoring at home, this method reduces the need for frequent clinic visits, thereby alleviating the burden on both patients and health care providers. Its portability makes it a practical option for diverse clinical settings, including those in remote or underserved areas. Furthermore, the potential to integrate this technology into telemedicine platforms enhances its utility, allowing clinicians to remotely monitor patient progress and provide timely interventions when necessary [8]. These findings align with previous reports emphasizing the need for reproducible, patient-centered measurement systems in chronic disease monitoring and further extend this concept to lymphedema care [14].

While this study has several strengths, including its prospective design and the inclusion of a diverse cohort of patients with upper and lower limb lymphedema, it is not without limitations. The relatively small sample size and the fact that the study was conducted at only 2 centers may limit the generalizability of the findings. Larger, multicenter studies are needed to confirm the results and assess the applicability of the 3D measurement bodysuit smartphone system across different populations and clinical settings. Additionally, the greater measurement errors observed at specific points, such as the base of the thigh and the wrist, suggest potential limitations in the system’s ability to accurately assess regions with complex anatomical contours or limited marker visibility. Future improvements to the technology should focus on addressing these challenges to further enhance its accuracy and reliability.

In conclusion, the 3D measurement bodysuit smartphone system represents a significant advancement in the field of lymphedema management. Its high accuracy, excellent reproducibility, and time efficiency make it a promising alternative to conventional manual methods. By providing access to accurate and user-friendly measurement tools, this system has the potential to transform lymphedema care by empowering patients to actively monitor their condition and enabling clinicians to deliver more efficient and personalized care. Future research should focus on addressing the system’s limitations, validating its use in broader and more diverse populations, and exploring its integration into telemedicine and advanced data analytics platforms.

## Data Availability

The datasets generated and analyzed during this study are available from the corresponding author on reasonable request. Due to patient privacy restrictions, data are not publicly available.

## Abbreviations

ISL: International Society of Lymphology
STROBE: Strengthening the Reporting of Observational Studies in Epidemiology
